# Diagnostic value of neutrophil to lymphocyte ratio and serum biomarkers in chronic osteomyelitis

**DOI:** 10.1038/s41598-025-05856-7

**Published:** 2025-07-01

**Authors:** Wenhui Zhao, Dongxiang Xu, Yanbin Dong, Wanwen Feng

**Affiliations:** 1 Department of Clinical Laboratory, The Affiliated Lianyungang Municipal Oriental Hospital of Kangda College of Nanjing Medical University, Lianyungang, 222042 China; 2 Department of Pathology, The Affiliated Lianyungang Hospital of Xuzhou Medical University, The First People’s Hospital of Lianyungang, Lianyungang, 222061 China; 3 Department of Orthopedic Surgery, The Affiliated Lianyungang Municipal Oriental Hospital of Kangda College of Nanjing Medical University, Lianyungang, 222042 China; 4 Center for Clinical Research and Translational Medicine, The Affiliated Lianyungang Municipal Oriental Hospital of Kangda College of Nanjing Medical University, Lianyungang, 222042 China; 5 Lianyungang Municipal Oriental Hospital, 57 Zhonghua West Road, Lianyun District, Lianyungang, 222042 China; 6 The First People’s Hospital of Lianyungang, 6 Zhenhua East Road, Haizhou District, Lianyungang, 222042 China

**Keywords:** Osteomyelitis, Biomarkers, Neutrophil to lymphocyte ratio, C-reactive protein, Tumor necrosis factor-alpha, Interleukin-6, Cell biology, Biomarkers

## Abstract

Chronic osteomyelitis (COM) is a persistent bone infection associated with severe complications, making early and accurate diagnosis essential. Traditional diagnostic methods, including imaging and bacterial cultures, are often limited by low sensitivity, long processing times, and the inability to detect infections in certain clinical scenarios. In this study, we evaluated the diagnostic utility of inflammatory biomarkers for COM, including neutrophil to lymphocyte ratio (NLR), C-reactive protein (CRP), tumor necrosis factor-alpha (TNF-α), and interleukin-6 (IL-6). A total of 200 participants, including 100 COM patients and 100 healthy controls, were enrolled. Our results showed that Gram-positive bacteria were more prevalent (59%), with *Staphylococcus aureus* being the most frequently isolated pathogen. Antibiotic resistance profiling revealed that Gram-positive bacteria exhibited high resistance to Penicillins but remained sensitive to Vancomycin and Linezolid. In contrast, Gram-negative bacteria showed high resistance to certain Penicillins, while sensitive to Carbapenems. Inflammatory marker levels (NLR, CRP, TNF-α, and IL-6) were significantly elevated in COM patients, with higher levels in Gram-negative infections. Multivariate logistic regression analysis and ROC curve analysis demonstrated that these inflammatory markers were significant predictors of COM, and the combination of these biomarkers showed superior diagnostic performance. Our findings suggest that NLR, CRP, TNF-α, and IL-6 are valuable diagnostic biomarkers for COM, and their combination enhances diagnostic precision, offering a promising tool for clinical management.

**Supplementary Information:**

The online version contains supplementary material available at 10.1038/s41598-025-05856-7.

## Introduction

Chronic osteomyelitis (COM) is a persistent infection of the bone and surrounding soft tissues, commonly arising as a complication of open fractures, hematogenous infections, or inadequately treated acute osteomyelitis^1-4^. COM can result in severe complications, including extensive bone destruction, osteonecrosis, and suppurative arthritis, which may result in amputation and long-term disability^5^. These outcomes can exert significant impact on a patient’s quality-of-life and increase the economic burden on their families. Recent studies reported that open fractures and implant-associated osteomyelitis account for 80% of all cases of osteomyelitis, with 10–30% progressing to chronic stages. Over the past four decades, the incidence of COM has almost doubled, reaching 21.8 per 100,000 individuals, with approximately 23% of patients requiring multiple debridement procedures and 6% ultimately needing amputation due to uncontrolled infections^6,7^. Due to its complex etiology, prolonged treatment course, high recurrence rates, and the potential for limb disability^8^it is critical that COM is diagnosed early and accurately to initiate timely interventions, improve patient prognosis, and prevent disease progression.

The current diagnostic approach for COM integrates clinical examination, imaging studies (e.g., X-rays, Computed Tomography (CT) scans), and bacterial cultures^8-10^. However, these tools are associated with a number of limitations. For example, the early-stage symptoms of COM are often non-specific, resembling other musculoskeletal disorders and contributing to diagnostic delays or errors. Furthermore, imaging techniques may fail to detect abnormalities in the early stages of infection, and significant structural changes are usually only observed in cases of advanced disease. Bacterial cultures, while considered the gold standard for identifying pathogens are frequently hindered by a range of factors, including previous antibiotic use, polymicrobial infections, or the presence of non-viable organisms^1,11^. Moreover, the lengthy processing time for cultures often delays treatment decisions, thus exacerbating patient outcomes.

These challenges highlight the urgent need for complementary diagnostic approaches. Recent advances in immunology have revealed that pathogen-associated molecular patterns (PAMPs) from both Gram-positive and Gram-negative bacteria activate Toll-like receptors (TLRs), particularly TLR2 and TLR4^11^. This activation triggers downstream signaling pathways such as NF-κB and MAPK, leading to the release of pro-inflammatory cytokines, including TNF-α and IL-6^12^^–^^15^. These cytokines, in turn, could serve as reliable biomarkers for diagnosing in COM. Moreover, serum inflammatory biomarkers, such as the NLR, CRP, TNF-α, and IL-6, have emerged as promising tools for the early detection and monitoring of infections^16-20^. Despite their widespread clinical use in diagnosing various conditions, their diagnostic utility in COM, particularly in distinguishing infections caused by Gram-positive and Gram-negative bacteria, remains underexplored.

In this study, we aimed to evaluate the diagnostic value of NLR, CRP, TNF-α, and IL-6 in COM patients, with a focus on their potential to enhance diagnostic precision and guide personalized treatment strategies. Specifically, we sought to address the gap in current understanding relating to how these biomarkers may be able to distinguish pathogen-specific infection profiles. By systematically analyzing these markers in COM patients and comparing them to healthy controls, we provide novel insights into their clinical applicability for early and accurate diagnosis.

## Materials and methods

### Patients and controls

The study included 100 patients diagnosed with COM treated at Lianyungang Municipal Oriental Hospital between January 2014 and June 2023. The cohort consisted of 58 males and 42 females. An additional 100 healthy individuals, undergoing routine physical examinations during the same period, were recruited as the control group. The sample size for this study was determined using the Power and Sample Size online tool (https://powerandsamplesize.com/), with the following parameters: a significance level (α) of 0.05, power (1 - β) set at 0.80 to ensure 80% probability of correctly rejecting the null hypothesis, and a 1:1 ratio for sample sizes between the two groups. The required sample size for each biomarker— NLR, CRP, TNF-α and IL-6 —was calculated based on mean values and standard deviations derived from literature and our preliminary data^21^. The study was approved by the Medical Ethics Committee of Lianyungang Municipal Oriental Hospital (Approval Number: 2022-030−01), and all participants provided written informed consent.

### Inclusion and exclusion criteria

The inclusion criteria required that patients had been diagnosed with COM, which was confirmed by X-ray or CT scans, in accordance with the criteria described in Practical Orthopedics^22^. Additionally, patients had to provide informed consent and have a positive bacterial culture from the lesion site identifying a single dominant strain of bacteria. Patients were excluded if they met the diagnostic criteria for COM but had negative bacterial cultures, had polymicrobial infections, were pregnant or lactating, or had a history of malignant tumors or serious infectious diseases. Additionally, patients with a history of antibiotic use within two weeks prior to admission were excluded from the study.

### Microbiological and antibiotic sensitivity testing

Upon admission, all COM patients underwent bacterial culture and antibiotic sensitivity testing. Exudates were collected from the lesion site using aseptic techniques. The procedure involved irrigating the sinus tract or puncture site with sterile physiological saline to collect the sample, which was then transported to the laboratory within two hours to ensure the integrity of the specimen. Bacterial cultures were incubated in a Thermo-240i carbon dioxide incubator (Thermo Fisher Scientific) under optimal conditions for 24–48 h. After incubation, bacterial colonies were analyzed using the MicroScan AutoSCAN-4 (Beckman Coulter) automated microbiological identification system, which identifies bacterial species based on their biochemical profiles. Antibiotic sensitivity testing was performed immediately following bacterial identification using the MicroScan AutoSCAN-4 system. The system tested various antibiotics against the isolated bacteria, providing precise information on the minimum inhibitory concentration (MIC) for each antibiotic. The results of the antibiotic sensitivity test were typically available within 48–72 h and used to guide the selection of the most appropriate antimicrobial therapy.

### Biomarker detection

All blood samples collected from COM patients were immediately processed after collection. The serum was separated by allowing the sample to stand at room temperature for at least 30 min, followed by centrifugation at 3500 rpm for 10 min, and then stored at −80 °C for further analysis. The samples were thawed only once before measurement to ensure the integrity of the biomarkers. For the healthy control group, blood collection, processing, and storage procedures were identical to those for the COM group to minimize diurnal variations in biomarker levels.

Upon admission, 4 ml of peripheral venous blood was drawn from COM patients on an empty stomach the following morning for the measurement of TNF-α and IL-6 levels using enzyme-linked immunosorbent assay (ELISA). The ELISA kits were supplied by Shanghai Enzyme-linked Biotechnology Co. Samples were processed according to the manufacturer’s instructions, and absorbance values were measured to determine the concentrations of TNF-α and IL-6. In addition, NLR and CRP levels were measured using the BC-5390 automated hematology analyzer (Shenzhen Mindray Bio-Medical Electronics Co.).

### Quality control

Microbiological testing and antibiotic sensitivity assays adhered to standardized protocols, with both positive and negative bacterial controls included in each batch. Regular calibration and maintenance of the MicroScan AutoSCAN-4 ensured optimal performance. For TNF-α and IL-6 detection, ELISA assays included controls, with the coefficient of variation (CV) maintained under 10% for precision. Detection limits followed manufacturer guidelines, and assays were performed in duplicate to reduce errors. NLR and CRP detection involved both internal and external quality controls. The BC-5390 analyzer underwent regular calibration, and quality control samples were tested alongside patient samples to ensure result accuracy, with automated checks ensuring consistent performance.

### Statistical methods

Statistical analyses were conducted using SPSS software (version 19.0). Quantitative data are expressed as the mean ± standard deviation (SD) or median (interquartile range, IQR), depending on the distribution. Normality was tested with the Shapiro-Wilk test. For normally distributed data, comparisons between the COM and control groups were performed using independent t-tests. For non-normally distributed data, comparisons were made using the Mann-Whitney U test. Categorical variables are presented as percentages and were compared using the chi-squared test.

A multivariate logistic regression model was constructed to determine the diagnostic value of biomarkers (NLR, CRP, TNF-α, and IL-6), adjusting for potential confounders such as age and sex. The diagnostic performance of individual biomarkers and their combinations was evaluated using ROC curve analysis. For the combined biomarkers, a composite score was derived by combining the selected biomarkers based on their weighted coefficients from the logistic regression model. The relative diagnostic performance of different biomarker combinations was compared by calculating the area under the curve (AUC) and assessing sensitivity and specificity. A statistical comparison of ROC curves was performed using the DeLong test to assess the significance of differences in the AUC between individual biomarkers and combinations^23^. Missing data were handled using listwise deletion (complete case analysis), as the proportion of missing data was minimal and occurred randomly, ensuring the integrity and reliability of the results. A P-value < 0.05 was considered statistically significant for all analyses.

## Results

### Clinical characteristics of the study cohorts

**Fig. 1:**
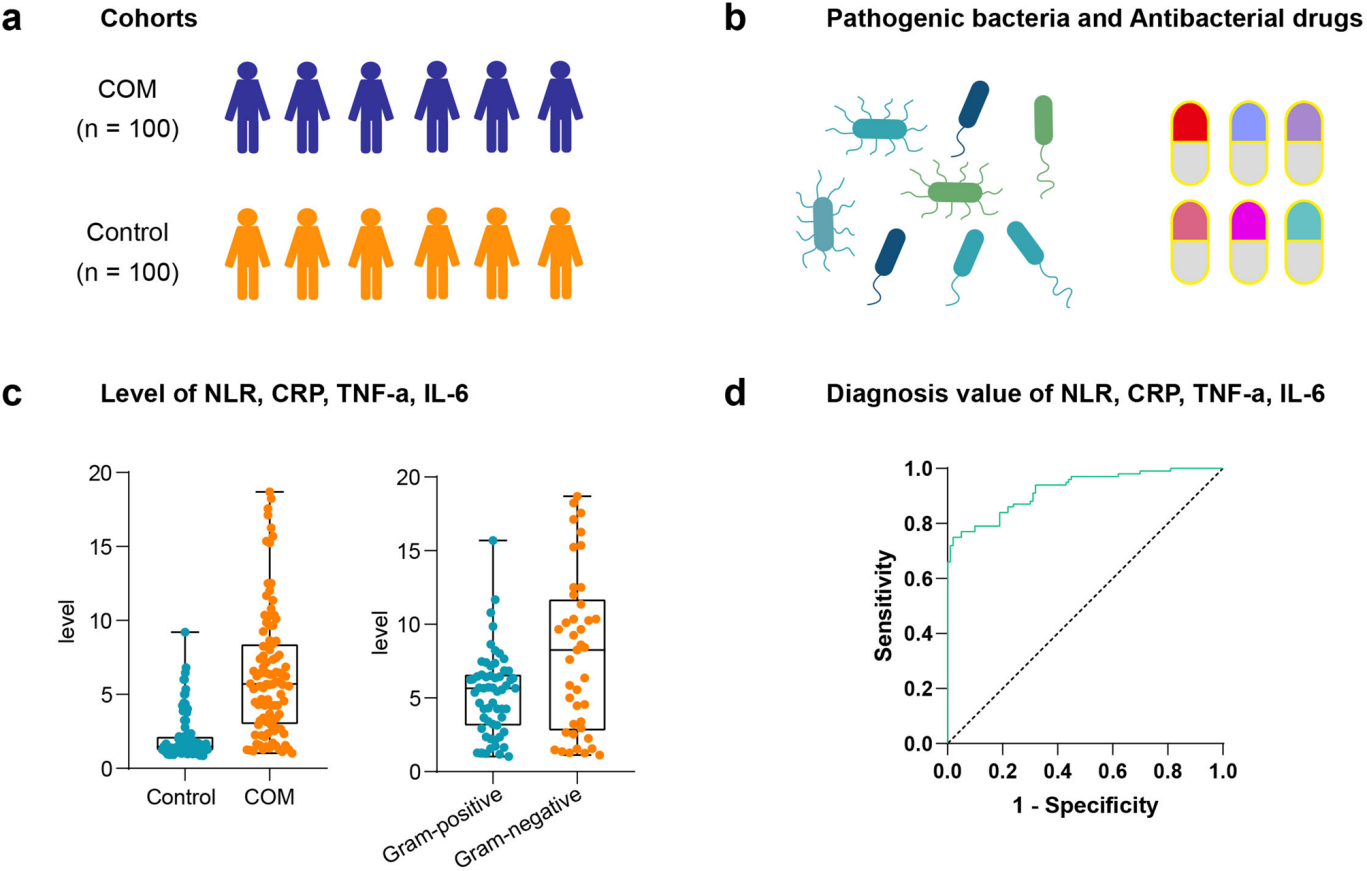
Study design and biomarker detection overview. **(a)** Diagram illustrating the two cohorts: 100 chronic osteomyelitis (COM) patients and 100 controls. **(b)** Illustration of the common pathogenic bacteria and antibacterial drugs used in the study. **(c)** Distribution of inflammatory marker levels, including neutrophil to lymphocyte ratio (NLR), C-reactive protein (CRP), tumor necrosis factor-alpha (TNF-α), and interleukin-6 (IL-6), between the control group and the COM group (left) and between Gram-positive and Gram-negative infection groups (right). **(d)** Receiver operating characteristic (ROC) curve showing the diagnostic value of inflammatory markers (NLR, CRP, TNF-α, and IL-6) for COM.

To investigate the pathogenic bacteria and antibacterial drug profiles associated with COM and assess the diagnostic value of key inflammatory markers, we enrolled 200 participants, including 100 COM patients and 100 healthy controls (Fig. 1). A range of clinical data, including sex, age, and lesion location, were acquired to ensure comparability between groups (Table 1). The two groups were well-matched, with no significant differences in sex distribution (male: 54% vs. 58%, *p* = 0.569) or mean age (42.1 ± 11.0 years vs. 43.4 ± 10.8 years, *p* = 0.415). Lesion distribution in COM patients varied, with the tibia (36%) and femur (28%) being the most frequently affected bones. These characteristics ensured the reliability of subsequent comparative analyses.

**Table 1:** Baseline characteristics of COM patients and control group. Comparison of demographic and clinical characteristics between the COM and control groups, including sex, age, and lesion location.

Characteristic		Control (*n* = 100)	COM (*n* = 100)	*P* value
Sex	Male	54	58	0.569
	Female	46	42	
Age, mean (SD), year		42.1 (11.0)	43.4 (10.8)	0.415
Lesion location	Tibia	/	36	
	Femur	/	28	
	Ulna and radius	/	9	
	Calcaneus	/	3	
	Tibiofibula	/	6	
	Jaw bone	/	3	
	Humerus	/	3	
	Others	/	12	

### Pathogen detection and distribution in COM patients

Given the infectious nature of COM, we investigated the distribution of pathogens to identify the predominant causative organisms (Table 2). Analysis confirmed that Gram-positive bacteria were more prevalent (59% of cases), with *Staphylococcus aureus* being the most frequently isolated pathogen (43%), followed by *Streptococcus viridans* (4%) and *Streptococcus pyogenes* (3%). However, Gram-negative bacteria also played a significant role (41%); *Pseudomonas aeruginosa* (13%) and *Escherichia coli* (12%) being the most common forms. In terms of demographic distribution, there were no significant differences between male and female patients regarding the isolation of Gram-positive and Gram-negative bacteria. Moreover, pathogen distribution did not show significant variation across different lesion locations, including tibia, femur, and other locations.

**Table 2:** Detection of pathogenic bacteria in patients with COM. Overview of the bacterial species identified in 100 COM patients. Gram-positive bacteria accounted for 59% of cases, with *Staphylococcus aureus* being the most common pathogen. Gram-negative bacteria made up 41% of isolates, with *Pseudomonas aeruginosa* and *Escherichia coli* being the most frequently identified species. The distribution of bacteria was also analyzed by gender and lesion location, with no significant differences observed between males and females or across different lesion sites. Statistical comparisons were performed between gender and lesion location categories using chi-squared tests.

Pathogenic bacteria	Number (*n* = 100)	Constituent ratio (%)	Gender			Lesion location			
			Male (*n* = 58)	Female (*n* = 42)	*P* value	Tibia (*n* = 36)	Femur (*n* = 28)	others (*n* = 36)	*P* value
Gram-positive bacteria	59	59							
*Staphylococcus aureus*	43	43	28	15	0.21	18	8	17	0.186
*Streptococcus viridans*	4	4	2	2	1	2	1	1	1
*Streptococcus pyogenes*	3	3	2	1	1	3	0	0	0.11
*Staphylococcus epidermidis*	5	5	3	2	1	2	1	2	1
Others	4	4	2	2	1	2	0	2	0.543
Gram-negative bacteria	41	41							
*Escherichia coli*	12	12	4	8	0.065	1	4	7	0.087
*Enterobacter cloacae*	4	4	2	2	1	2	1	1	1
*Pseudomonas aeruginosa*	13	13	10	3	0.138	4	7	2	0.078
*Klebsiella pneumoniae*	8	8	1	7	0.009	2	3	3	0.903
Others	4	4	4	0	0.137	0	3	1	0.091

### Antibiotic resistance profile of major pathogenic bacteria

To guide effective antibiotic therapy, we conducted resistance profiling of major pathogenic bacteria. For Gram-positive bacteria (Table 3), including *Staphylococcus aureus*,* Streptococcus viridans*,* Streptococcus pyogenes*, and *Staphylococcus* epidermidis, high resistance rates (97.7–100%) were observed against Penicillins such as Penicillin and Ampicillin, with resistance also notable against Erythromycin. However, these strains were sensitive to Glycopeptides (Vancomycin) and Oxazolidinones (Linezolid). In contrast, for Gram-negative bacteria (Table 4), resistance varied significantly among different species. *Escherichia coli*,* Enterobacter cloacae*,* Pseudomonas aeruginosa*, and *Klebsiella pneumoniae* exhibited high resistance to Penicillins such as Ampicillin and Piperacillin, with resistance rates up to 92.3%. Interestingly, these Gram-negative strains remained sensitive to Carbapenems (Meropenem and Imipenem), with low resistance rates, highlighting their potential utility as therapeutic options.

**Table 3:** Antibiotic resistance profile of Gram-positive bacteria. Analysis of resistance rates of *Staphylococcus aureus*,* Streptococcus viridans*,* Streptococcus pyogenes*, and *Staphylococcus epidermidis* against various antibiotics. P value indicates the statistical comparison of resistance rates between different strains of Gram-positive bacteria.

Antibiotic families	Antibacterial drugs	Staphylococcus aureus (*n* = 43)		Streptococcus viridans (*n* = 4)		Streptococcus pyogenes (*n* = 3)		Staphylococcus epidermidis (*n* = 5)		*P* value
		No. of resistant strains	Resistant rate (%)	No. of resistant strains	Resistant rate (%)	No. of resistant strains	Resistant rate (%)	No. of resistant strains	Resistant rate (%)	
**Penicillins**	Penicillin	42	97.7	4	100	3	100	5	100	0.06
	Ampicillin	43	100	4	100	3	100	4	80	< 0.001
**Macrolides**	Erythromycin	28	65.1	3	75	2	66.7	4	80	< 0.001
**Tetracyclines**	Tetracycline	6	14	1	25	2	66.7	3	60	< 0.001
**Lincomycins**	Lincomycin	41	95.3	2	50	1	33.3	3	60	< 0.001
**Quinolones**	Levofloxacin	18	41.9	2	50	2	66.7	4	80	< 0.001
	Ciprofloxacin	18	41.9	2	50	2	66.7	3	60	< 0.001
	Moxifloxacin	8	18.6	1	25	1	33.3	2	40	< 0.001
**Cephalosporins**	Cefoperazone	24	55.8	1	25	1	33.3	1	20	< 0.001
**Glycopeptides**	Vancomycin	0	0	0	0	0	0	0	0	> 0.999
**Oxazolidinones**	Linezolid	0	0	0	0	0	0	0	0	> 0.999

**Table 4:** Antibiotic resistance profile of Gram-negative bacteria. Analysis of resistance rates of *Escherichia coli*,* Enterobacter cloacae*,* Pseudomonas aeruginosa*, and *Klebsiella pneumoniae* against various antibiotics. P value indicates the statistical comparison of resistance rates between different strains of Gram-negative bacteria.

Antibiotic families	Antibacterial drugs	*Escherichia coli* (*n* = 12)		Enterobacter cloacae (*n* = 4)		Pseudomonas aeruginosa (*n* = 13)		Klebsiella pneumoniae (*n* = 8)		*P* value
		No. of resistant strains	Resistant rate (%)	No. of resistant strains	Resistant rate (%)	No. of resistant strains	Resistant rate (%)	No. of resistant strains	Resistant rate (%)	
**Carbapenems**	Meropenem	0	0	0	0	1	7.7	1	12.5	< 0.001
	Imipenem	1	8.3	0	0	1	7.7	1	12.5	< 0.001
**Cephalosporins**	Cefepime	3	25	0	0	0	0	2	37.5	< 0.001
	Cefotaxime	4	33.3	2	50	3	23.1	2	25	< 0.001
**Aminoglycosides**	Amikacin	1	8.3	0	0	1	7.7	1	12.5	< 0.001
	Gentamicin	2	16.7	0	0	2	15.4	1	12.5	< 0.001
	Tobramycin	2	16.7	0	0	2	15.4	1	12.5	< 0.001
**Quinolones**	Ciprofloxacin	7	58.3	1	25	2	15.4	3	37.5	< 0.001
	Levofloxacin	6	50	1	25	4	30.8	2	25	< 0.001
**Penicillins**	Ampicillin	10	83.3	3	75	12	92.3	7	87.5	0.009
	Piperacillin	9	75	2	50	6	46.2	4	50	< 0.001

### Inflammatory marker levels in COM and pathogen-specific differences

**Fig. 2:**
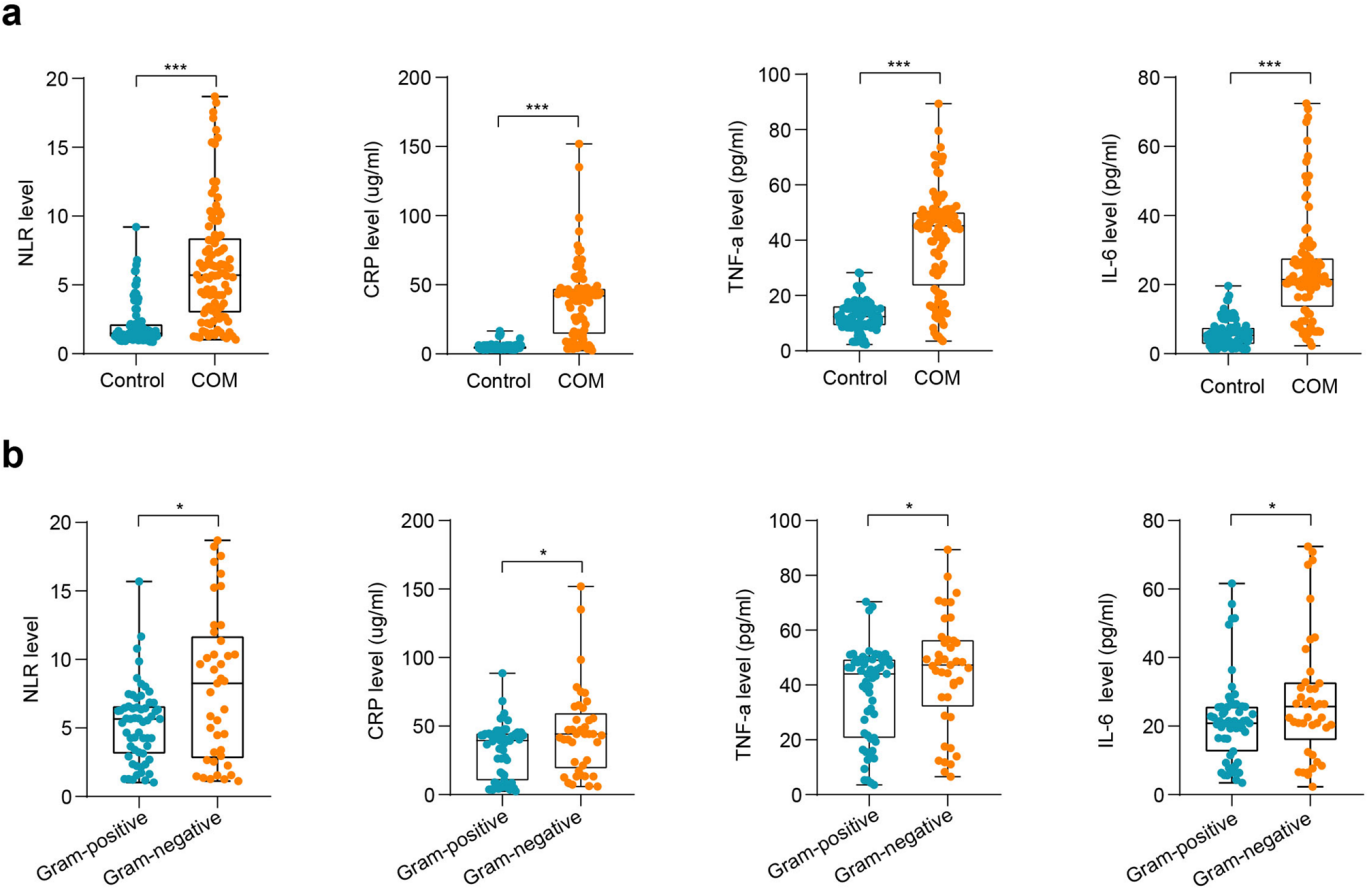
Inflammatory marker levels in COM and pathogen-specific groups. **(a)** Comparison of NLR, CRP, TNF-α, and IL-6 levels between COM patients and healthy controls. All markers were significantly elevated in the COM group. **(b)** Inflammatory marker levels stratified by Gram-positive and Gram-negative bacterial infections. Gram-negative infections were associated with higher levels of all markers, indicating a stronger inflammatory response. Data in a and b are presented as the mean ± SD for normally distributed data and as the Median (IQR) for non-normally distributed data. *p < 0.05, ***p < 0.001.

Since COM is associated with a pronounced inflammatory response, we measured the levels of key inflammatory markers (NLR, CRP, TNF-α, and IL-6) in both COM patients and healthy controls to evaluate their diagnostic relevance. Analysis revealed that all four markers were significantly elevated in COM patients when compared to controls, as shown in Fig. 2a. Further analysis revealed pathogen-specific differences in inflammatory marker levels, as shown in Fig. 2b. Infections caused by Gram-negative bacteria were associated with significantly higher levels of NLR, CRP, TNF-α, and IL-6 compared to Gram-positive infections (*P* < 0.05). These data suggest that Gram-negative pathogens may trigger a more robust inflammatory response, which could have implications for the severity of disease and patient management. Additionally, subgroup analysis based on lesion location revealed no significant changes in the levels of these inflammatory markers between the different lesion sites (Table S1), indicating that the inflammatory response in COM is not significantly affected by lesion location.

### Diagnostic value of inflammatory markers for COM

**Fig. 3:**
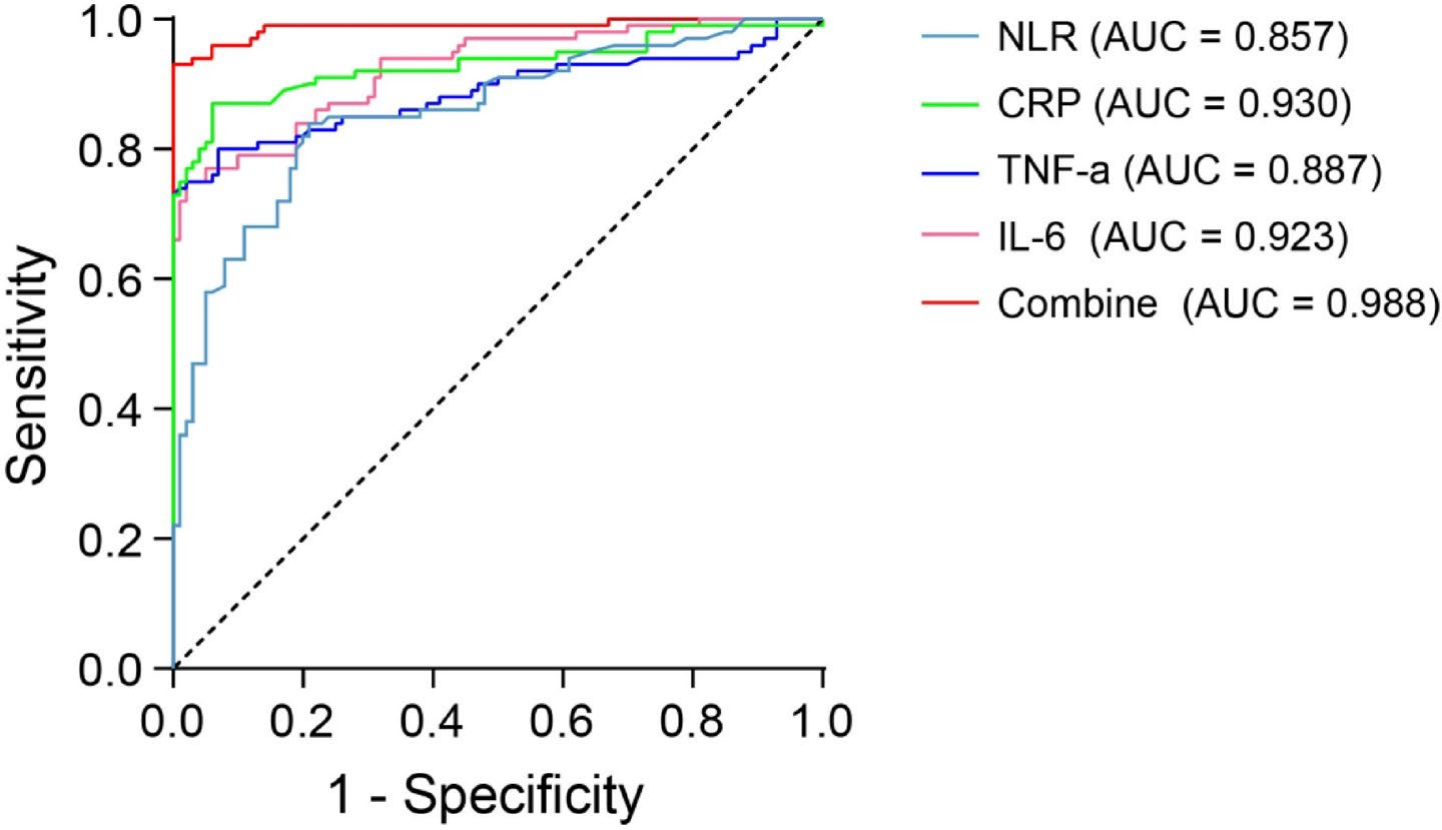
ROC curve analysis showing the diagnostic performance of inflammatory markers. ROC curves showing the diagnostic performance of individual inflammatory markers (NLR, CRP, TNF-α, IL-6) and their combination.

To further evaluate the diagnostic relevance of inflammatory biomarkers in COM, a multivariate logistic regression analysis was performed, adjusting for gender and age. The results showed that NLR (OR = 1.86), CRP (OR = 1.34), TNF-α (OR = 1.17), and IL-6 (OR = 1.32) were all significant predictors of COM, as demonstrated in Table 5, indicating strong associations (*P* < 0.001). Additionally, ROC curve analysis was used to assess the sensitivity and specificity of these markers both individually and in combination (Table 6; Fig. 3). Among the individual markers, CRP exhibited the highest diagnostic accuracy with an AUC of 0.930, followed closely by IL-6 (AUC = 0.923) and TNF-α (AUC = 0.887). NLR demonstrated a moderate diagnostic value with an AUC of 0.857. Remarkably, the combination of these biomarkers achieved an AUC of 0.988, which was significantly higher than that of any individual marker (*P* < 0.001), indicating that a multi-marker approach offers superior diagnostic performance compared to single biomarkers.

**Table 5:** Multivariate logistic regression analysis of inflammatory markers for COM diagnosis. Results of the multivariate logistic regression analysis for inflammatory markers (NLR, CRP, TNF-α, and IL-6) in COM, adjusted for age and gender. OR, 95% CI, and P values are shown for each biomarker.

Variable	Total (*n*)	OR (95% CI)	*P* value
NLR	200	1.86 (1.55–2.23)	< 0.001
CRP (µg/ml)	200	1.34 (1.19–1.52)	< 0.001
TNF-α (pg/ml)	200	1.17 (1.11–1.22)	< 0.001
IL6 (pg/ml)	200	1.32 (1.22–1.43)	< 0.001

**Table 6:** Diagnostic performance of inflammatory markers in COM. Diagnostic performance of individual biomarkers (NLR, CRP, TNF-α, IL-6) and their combination for COM, including cut-off values, sensitivity, specificity, positive predictive value (PPV), negative predictive value (NPV), area under the curve (AUC), and 95% confidence intervals (CI) are provided for each marker. The P values represent the statistical comparison between the ROC curves for individual biomarkers and their combination.

Variable	Cut-off values	Sensitivity	Specificity	PPV	NPV	AUC	95% CI	*P* value
NLR	2.185	0.840	0.790	0.8	0.832	0.857	0.804–0.909	< 0.001
CRP (µg/ml)	7.065	0.870	0.940	0.935	0.878	0.930	0.891–0.969	< 0.001
TNF-α (pg/ml)	25.37	0.8	0.930	0.919	0.823	0.887	0.836–0.938	< 0.001
IL-6 (pg/ml)	5.920	0.750	0.980	0.974	0.797	0.923	0.887–0.959	< 0.001
Combination	-	0.930	1	1	0.935	0.988	0.974–1	< 0.001

## Discussion

In this study, we demonstrated that NLR, CRP, TNF-α, and IL-6 levels were significantly elevated in COM patients, thus highlighting their potential as diagnostic biomarkers. The combination of multiple markers further improved diagnostic performance, emphasizing the value of a multi-marker approach. Additionally, Gram-negative infections were associated with higher levels of NLR, TNF-α, and CRP than Gram-positive infections. This reflects distinct inflammatory responses and highlights the need for tailored diagnostics. Our findings confirm the predominance of *Staphylococcus aureus* in COM infections and provide valuable insights into optimizing early diagnosis and personalized treatment strategies for COM.

Our analysis showed that the tibia and femur were the most frequently affected bones in COM, with lower-limb involvement accounting for over 50% of cases. This finding aligns with previous results of Yeh et al. who reported that COM predominantly affects the lower limbs due to their higher exposure to trauma and compromised vascular supply^24^. These anatomical and physiological vulnerabilities may contribute to the development of chronic infections in these regions. In this study, pathogen detection indicated that Gram-positive bacteria were the leading causative agents, accounting for 59% of cases. *Staphylococcus aureus* identified as the most prevalent pathogen (43%). These findings align closely with previous reports^25,26^which consistently identified *Staphylococcus aureus* as the predominant pathogen in osteomyelitis. The presence of other Gram-positive bacteria, such as *Streptococcus viridans* and *Streptococcus pyogenes*, emphasized the diversity of the pathogens contributing to bone infections. Furthermore, Gram-negative bacteria, including *Pseudomonas aeruginosa* and *Escherichia coli*, were frequently identified. This highlights the need for broad-spectrum coverage during empirical treatment.

The antibiotic resistance profile of major pathogenic bacteria in our study highlights a significant clinical challenge in the management of COM. For Gram-positive bacteria, high resistance rates were observed against Penicillins, such as Penicillin and Ampicillin, along with notable resistance to Erythromycin. However, these strains exhibited sensitivity to Vancomycin and Linezolid. This confirms their efficacy as therapeutic options for treating infections caused by *Staphylococcus aureus*,* Streptococcus viridans*,* Streptococcus pyogenes*, and *Staphylococcus epidermidis*. This finding aligns with regional resistance patterns, where Gram-positive pathogens are increasingly resistant to commonly used antibiotics^27^. In contrast, for Gram-negative bacteria, resistance varied significantly across species. *Escherichia coli*,* Enterobacter cloacae*,* Pseudomonas aeruginosa*, and *Klebsiella pneumoniae* showed high resistance to Penicillins such as Ampicillin and Piperacillin, with resistance rates reaching up to 92.3%. Interestingly, these Gram-negative strains remained sensitive to Carbapenems (Meropenem and Imipenem), with low resistance rates. This highlights the potential utility of Carbapenems as a therapeutic option. The observed differences in resistance profiles between Gram-positive and Gram-negative bacteria underscore the importance of understanding pathogen-specific resistance patterns when selecting antibiotics for treatment. This finding is consistent with global concerns about the rise of multidrug-resistant bacterial pathogens, commonly known as superbugs, which pose an increasing threat to public health^28^.

Inflammatory markers play crucial roles in the immune response, highlighting these as valuable tools for the clinical diagnosis of infectious diseases^29,30^. Research has shown that PAMPs activate TLRs on immune cells, playing a key role in pathogen recognition. Specifically, TLR2 recognizes lipoteichoic acid from Gram-positive bacteria, while TLR4 is activated by lipopolysaccharide (LPS) from Gram-negative bacteria^12^. This activation triggers a series of downstream signaling pathways, leading to the excessive release of pro-inflammatory cytokines^13^. The abnormal activation of immune cells and the excessive release of inflammatory mediators are key features in the pathogenesis of COM^31^. For example, infections caused by *Staphylococcus aureus* can stimulate macrophages *via* endotoxins, triggering the release of cytokines such as TNF-α and IL-6. These cytokines activate vascular endothelial cells, promoting the expression of adhesion molecules and contributing to bone tissue destruction^32^. Given the pathophysiology of COM, the selection of appropriate inflammatory markers for early diagnosis is essential for guiding timely interventions.

Among commonly used clinical biomarkers, NLR, CRP, TNF-α, and IL-6 are particularly valuable for evaluating inflammatory responses. NLR, which reflects changes in the distribution of leukocytes, has gained significant clinical attention due to its ease of measurement, low cost, and high sensitivity. Elevated levels of NLR have been linked to poor outcomes in various inflammatory diseases, including acute pancreatitis and ulcerative colitis, and are known to be predictive of disease severity^33,34^. Similarly, CRP, an acute-phase protein synthesized in the liver, is known to increase significantly during bacterial infections but remains relatively low in viral infections. This makes CRP a sensitive and specific indicator of bacterial infections^17,35^. CRP is also commonly utilized to monitor the severity and recurrence of infectious diseases, including COM. TNF-α, a pro-inflammatory cytokine, is crucial in COM progression. Its levels correlate with disease severity^36,37^. Likewise, IL-6 activates immune cells and endothelial function, contributing to tissue damage^32^. Both TNF-α and IL-6 increase in bacterial infections, highlighting their diagnostic value in COM^38^.

In this study, we found that the levels of NLR, CRP, TNF-α, and IL-6 were significantly elevated in COM patients compared to healthy controls, demonstrating their diagnostic potential. Multivariate logistic regression and ROC curve analysis confirmed the value of these biomarkers. CRP showed the highest individual diagnostic accuracy. A previous meta-analysis, utilizing Forrest plots and ROC curves, aligned with previous findings. The meta-analysis also highlighted that CRP exhibited high sensitivity and specificity for diabetic foot osteomyelitis, with pooled values of 0.72 and 0.76, respectively, and a pooled AUC of 0.77^39^. This variation in AUC may reflect differences in inclusion criteria and control groups. Moreover, the levels of TNF-α and IL-6 are elevated not only in COM but also in other chronic inflammatory conditions like psoriatic arthritis (PsA) and rheumatoid arthritis (RA). In RA, TNF-α has a diagnostic value of 0.99, while IL-6 is 0.67 (compared to PsA)^40^. These biomarkers also show high expression and diagnostic value in other conditions, suggesting their broad applicability. Future studies should evaluate their specificity in both COM and other inflammatory conditions. This would help establish more robust and condition-specific cut-off values for optimal diagnostic accuracy. The combination of NLR, CRP, TNF-α, and IL-6, achieving an AUC of 0.988, underscores the complementary roles of cellular immunity (NLR), acute-phase proteins (CRP), and cytokine networks (TNF-α and IL-6) in capturing the multidimensional inflammatory landscape of COM. Furthermore, the elevated levels of these biomarkers in patients with Gram-negative infections, compared to Gram-positive infections, align with previous studies^41^. This elevation corresponds with the hyperactivation of innate immune pathways, particularly TLR4-mediated signaling. For example, Gram-negative pathogens such as *Pseudomonas aeruginosa* and *Escherichia coli* primarily activate TLR4 *via* LPS recognition. This leads to robust NF-κB activation and the subsequent release of IL-6 and TNF-α. This mechanistic pathway is further supported by studies on COVID-19^15^, where TLR4 hyperactivation contributes to cytokine storms, a phenomenon that mirrors the systemic inflammation observed in our Gram-negative COM cohort.

The clinical utility of these biomarkers lies in their ability to provide accessible, rapid, and cost-effective diagnostic information. We estimate that the combined cost for testing the four biomarkers is approximately $20. The dynamics of these biomarkers during antibiotic therapy further support their clinical utility. Studies have shown that in diabetic foot osteomyelitis, CRP and IL-6 levels significantly declined after three weeks of vancomycin/piperacillin-tazobactam therapy^42^. Another study indicated that after initiating antibiotic treatment, WBC, CRP, and PCT levels returned to near-normal levels by day 7, although ESR remained elevated in COM until 3 months post-treatment^43^. This suggests that the kinetics of NLR, CRP, TNF-α, and IL-6 may be more effective for predicting early treatment response. In scenarios involving delayed or negative bacterial cultures, these biomarkers could provide critical diagnostic support, enabling timely and appropriate intervention. Incorporating NLR, CRP, TNF-α, and IL-6 into routine diagnostic protocols for COM holds significant promise for improving diagnostic accuracy, guiding personalized treatment strategies, and ultimately enhancing patient outcomes.

### Limitations

This study offers valuable insights into the diagnostic utility of NLR, CRP, TNF-α, and IL-6 for COM, but several limitations should be acknowledged. First, comorbidities, such as diabetes and autoimmune disorders, may affect biomarker levels. Research has shown that inflammation markers, such as CRP, TNF-α, and IL-6 are significantly elevated in conditions such as Type 2 diabetes mellitus^44^. Future studies should address this by conducting more detailed subgroup analyses based on patients’ medical histories, providing a deeper understanding of the factors influencing biomarker levels. Second, the lack of disease control groups is a significant limitation. Biomarker levels in other bone and joint inflammatory conditions, such as RA and osteoarthritis (OA), have shown marked elevations in CRP and TNF-α^45,46^. However, studies have reported that IL-6 levels do not significantly change in conditions like chronic recurrent multifocal osteomyelitis and juvenile idiopathic arthritis^47^. Comparing these biomarkers across different inflammatory diseases will be crucial for validating their specificity and diagnostic potential and improving the clinical applicability of our findings. In future studies, we plan to include patients with RA and OA to assess the specificity of these biomarkers. Third, the absence of stratification by infection etiology, clinical type, infection stage, and disease severity limits our understanding of how biomarkers perform across different infection profiles. Stratified analyses by these factors in future research will refine the diagnostic use of these biomarkers. Fourth, the retrospective nature of the study introduces biases, including selection bias and the inability to control for unmeasured confounders, which could affect generalizability. We acknowledge these limitations and recommend that future prospective studies help address these issues. Fifth, the generalizability of our findings is limited by the relatively homogenous patient population studied. Future studies should validate these results in diverse patient groups and across different geographic locations to assess the broader applicability of our findings. Sixth, this study did not compare the biomarkers we evaluated with other commonly used biomarkers, such as WBC count or ESR, which are typically part of the diagnostic workup for infections. This comparison will be crucial to assess their relative diagnostic value. Lastly, we observed two cases of S. epidermidis infection, which may indicate superficial contamination despite aseptic procedures, emphasizing the need for stringent sampling methods. Expanding the sample size and exploring other biomarkers, such as procalcitonin and interleukin-1, may improve diagnostic accuracy. Longitudinal studies to assess biomarker levels throughout treatment will also enhance our ability to monitor disease progression and predict treatment outcomes.

## Conclusions

Our analysis demonstrated that NLR, CRP, TNF-α, and IL-6 are valuable diagnostic markers for COM. The combination of these markers enhances diagnostic precision, thus providing practical tools for early detection, especially when bacterial cultures are delayed or inconclusive. The distinct biomarker profiles observed between Gram-positive and Gram-negative infections underscore the potential for etiology-driven therapeutic strategies, aligning with pathogen-specific antibiotic resistance patterns. Importantly, the preserved sensitivity of key antibiotics against predominant pathogens supports the integration of biomarker-guided early intervention to optimize empirical therapy and mitigate antimicrobial resistance. Incorporating these accessible biomarkers into routine clinical practice could facilitate timely intervention and personalized treatment. Future research with larger cohorts and additional biomarkers will further refine diagnostic strategies and improve patient outcomes in COM.

## Electronic supplementary material

Below is the link to the electronic supplementary material.

Supplementary Material 1

## Data Availability

All data generated or analysed during this study are included in this published article.
