# Supplementary material for: Diagnostic value of neutrophil to lymphocyte ratio and serum biomarkers in chronic osteomyelitis
Source: Sci Rep. 2025 Jul 1;15:21752. doi: 10.1038/s41598-025-05856-7 (PMC12216443; doi:10.1038/s41598-025-05856-7)
Supplement: Supplementary file 1 — Supplementary Material 1 [file 41598_2025_5856_MOESM1_ESM.docx]

**Table S1. Inflammatory marker levels stratified by lesion location in COM.** The levels of inflammatory markers (NLR, CRP, TNF-α, and IL-6) stratified by lesion location (Tibia, Femur, and others) in COM. The median (IQR) for NLR and CRP and the mean (SD) for TNF-α and IL-6 are provided for the total group, as well as for patients stratified by lesion location. P values represent comparisons of the inflammatory marker levels between the different lesion location groups.

| **Characteristic** | **Total**  **(n = 100)** | **Tibia**  **(n = 36)** | **Femur**  **(n = 28)** | **others**  **(n = 36)** | ***P* value** |
| --- | --- | --- | --- | --- | --- |
| Age, mean (SD), year | 43.4 (10.8) | 42.2 (11.0) | 42.2 (11.8) | 45.5 (9.8) | 0.34 |
| gender, n (%) |  |  |  |  | 0.63 |
| Male | 58 (58.0) | 19 (52.8) | 16 (57.1) | 23 (63.9) |  |
| Female | 42 (42.0) | 17 (47.2) | 12 (42.9) | 13 (36.1) |  |
| NLR, Median (IQR) | 5.7 (3.1, 8.3) | 6.3 (4.2, 8.9) | 5.3 (3.1, 9.8) | 5.6 (1.7, 6.5) | 0.091 |
| CRP, Median (IQR),  (μg/ml) | 42.0 (15.0, 46.8) | 42.6 (23.0, 46.9) | 41.9 (8.9, 47.2) | 40.2 (15.0, 44.8) | 0.836 |
| TNF-α, mean (SD), (pg/ml) | 40.1 (18.8) | 42.6 (19.5) | 40.9 (22.4) | 37.1 (14.9) | 0.459 |
| IL6, mean (SD), (pg/ml) | 24.1 (18.8) | 27.3 (17.4) | 21.3 (11.7) | 23.1(15.7) | 0.28 |
